# 671 Multidisciplinary Outpatient Rounds: A New Approach to Optimize Pediatric Burn Care in the Outpatient Setting

**DOI:** 10.1093/jbcr/iraf019.300

**Published:** 2025-04-01

**Authors:** Chinaemelum Akpunonu, Sarah Shpil, Emily Haughawout, Jacqueline White, Myra Gray, Renata Fabia, Dana Schwartz, Rajan Thakkar

**Affiliations:** Burn Center at Nationwide Children’s Hospital; Burn Center at Nationwide Children’s Hospital; Burn Center at Nationwide Children’s Hospital; Burn Center at Nationwide Children’s Hospital; Nationwide Children’s Hospital; Nationwide Children’s Hospital; Nationwide Children’s Hospital; Nationwide Children’s Hospital

## Abstract

**Introduction:**

Multidisciplinary care and effective communication are essential to pediatric burn management. Inpatient multidisciplinary rounds are a requirement for American Burn Association (ABA) verification, as they allow for holistic care and address barriers to recovery and discharge. The ABA does not mandate such rounds for outpatient care. However, recognizing the equal importance of coordinating complex outpatient care and the need for cross-team communication, our center created weekly outpatient multidisciplinary rounds to improve care delivery.

**Methods:**

A one-hour weekly multidisciplinary outpatient burn meeting was created to improve communication and efficiency of care. The rounds consisted of physical therapists (PT), occupational therapists (OT), social workers, burn clinic nurses, psychologists, advanced practice providers, and surgeons. A database was created to record the care interventions made during these rounds. The changes were categorized into 3 domains: PT/OT considerations, psychosocial needs, and coordination of care. A retrospective review of outpatients seen between January 2023 to August 2024 was conducted and analyzed.

**Results:**

Two hundred fifty-six patients were identified with 6 excluded due to incomplete data. One hundred and forty patients (56%) were identified as needing additional PT/OT services, with added visit time for PT/OT to measure for garments being the most common (48%). Fifty-one patients (20.4%) were noted to have at least one psychosocial need that was detected during the rounds. Under this domain, 29 patients (11.6%) had missed appointments for various reasons and required additional outreach. Transportation barriers were the second highest psychosocial need identified (4.8%). Sixty-eight patients (27.2%) required further coordination of care efforts. Among these, 22 (32%) were scheduled with a different provider to improve efficacy.

**Conclusions:**

This work highlights the complexity of caring for outpatient pediatric burn patients and their families. Attending clinic visits places a substantial burden on patients’ families, in terms of both resources and time. Bringing together the entire multidisciplinary team in weekly outpatient rounds allowed us to identify each patient’s unique needs prior to the visit and deliver optimal and efficient care.

**Applicability of Research to Practice:**

Effective and efficient communication among the burn team must occur to address the diverse challenges and barriers that patients and their families have. A weekly multidisciplinary outpatient meeting improves communication, efficiency, and may improve short- and long-term outcomes.

**Funding for the Study:**

N/A

